# The Book World of Medicine and Science

**Published:** 1906-05-26

**Authors:** 


### THE BOOK WORLD OF MEDICINE AND SCIENCE.

THE SACRED TENTH; OR STUDIES IN TITHE-GIVING, ANCIENT AND MODERN. By HENRY LANSDELL, D.D., F.R.G.S., M.R.A.S., Chaplain of Modern College, Blackheath. Author of "Through Siberia," "Russian Central Asia," "Chinese Central Asia," etc. With Portrait, Maps, Illustrations, and Appendices, containing a Bibliography on Tithegiving, Lists of Crown Grantees and Modern Owners of English Alienated Tithes, etc. Two volumes. (London: Society for Promoting Christian Knowledge, Northumberland Avenue, W.C.)

IF Dr. Lansdell's book has a fault it is that there is too much of it; it cannot be expected that these two bulky volumes will reach the mass of the people, and yet it is eminently desirable that they should do so, and that our nation should learn again the old and long-forgotten duty of systematic giving to the cause of religion and charity. Nevertheless, basing the claim for the dedication of the "Sacred Tenth" of every man's possessions to God on history, Dr. Lansdell does well to point out how in all ages and among all peoples, Christian, Jewish, and heathen, custom and religious command insisted on the setting aside of God's portion before man began to enjoy his own share. The history of tithegiving, necessarily long, is both interesting and convincing. Dr. Lansdell admits that in the Levitical times the tithe went for more than religion and charity. To the hands of the Levites was left the administration of justice, and the provision for the poor, national education, the care of the Scriptures, as well as the cost of maintaining public worship, were all met out of the compulsory tenth. Thus the modern man may claim that in paying his rates and taxes he is paying his tithe; but it is more than doubtful if these claims take up a tenth part of any man's income. Dr. Lansdell does not, however, insist on every man paying what he calls the "decimal of duty" either to religion or charity. His aim is rather to impress upon his readers the duty of giving systematically and regularly a proportion of income fixed with due regard to the other claims upon the purse. Casual giving is apt to degenerate into no giving at all. The record of the English nation is far from satisfactory. It is reckoned that not more than one per cent. of the income of the nation is given to religion and philanthropic objects. It is, of course, impossible to estimate what is given privately and never comes into published subscription lists, but experience largely shows that those who do not give to well-known objects do not often give lavishly to private needs. Dr. Lansdell points out that while appeals for charity are common enough, they are often too vague and general, and do not call upon people to give either a fixed sum or a fixed proportion of their goods to the object in need of help. They depend therefore on their immediate effect, and however successful that may be, it does not, in the end, do so much as the habit of regular giving, which, moreover, being spread over the whole year, is not felt as so great a tax as a relatively large sum at one time. There are in America several societies for the encouragement of proportionate giving, and a beginning has been made here, but the movement is still in its infancy, and it is sincerely to be hoped that Dr. Lansdell will be able to give it the needed impetus, either by means of this invaluable book, or by some less exhaustive and more handy treatise.

